# Marginal diversity analysis of conservation of Chinese domestic duck breeds

**DOI:** 10.1038/s41598-019-49652-6

**Published:** 2019-09-11

**Authors:** Yang Zhang, Laidi Wang, Youqing Bian, Zhaoshan Wang, Qi Xu, Guobin Chang, Guohong Chen

**Affiliations:** 1 College of Animal Science and Technology, Yangzhou University, Yangzhou, 225009 Jiangsu People’s Republic of China; 2 Jiangsu Sci-tech Demonstration Garden of Modern Animal Husbandy, Taizhou, 225300 Jiangsu People’s Republic of China; 3 Jiangsu Eco Food Company Limited, Suqian, 223600 Jiangsu People’s Republic of China

**Keywords:** Genetics, Predictive markers

## Abstract

The present study aimed to systematically evaluate the genetic diversity of Chinese domestic duck breeds and ensure the most effective allocation and usage of conservation funds. We first performed an analysis of DNA genetic distance in 21 duck breeds by measuring short tandem repeats. Then, we calculated the extinction probability, contribution rate, and marginal diversity for each breed. The results showed that the extinction rate of the Zhongshan duck, Guangxi duck, and Ji’an duck were the highest at 0.67, 0.59, and 0.59, respectively, and that of the Linwu duck, Jinding duck, and Gaoyou duck were the lowest at 0.15, 0.18, and 0.19, respectively. The current diversity of populations was 7.72 and the expected diversity in five hundred years is 5.14 ± 1.15. The marginal diversity of the Chinese Muscovy duck was the largest (−2.20), accounting for 42.61% of the expected diversity, followed by the Guangxi duck (−0.49, 9.44%), whereas the Jinding duck was the smallest (−0.12; 2.32%). The protection potency of the Chinese Muscovy duck was the largest (0.61), followed by Guangxi duck (0.29), whereas the Jinding duck was the smallest (0.02). This study provides a reference for determining the conservation priority of Chinese domestic duck breeds or genetic resources.

## Introduction

Animal genetic resources are the basis of the sustainable development of animal husbandry^1,2^, and China is one of the foremost countries in this respect, accounting for one-sixth of the world’s animal genetic resources^3,4^. Two systematic and comprehensive surveys were conducted on animal genetic resources: one from 1970 to 1980, and the other in 2004. According to the Report on Domestic Animal Genetic Resource in China (edited in 2012)^5^, 777 breeds of animal genetic resources have been formally named in China, including 556 local breeds, 109 cultivated breeds, 104 introduced breeds, and eight other breeds. With respect to breeds of poultry, there are 116 chickens, 34 ducks, 31 geese, three turkeys, three pigeons, and two species of quail.

However, animal genetic resources have shown an overall decline in China since 1970s, due to unknown resources in some areas, low *in vivo* conservation, loss of animal genetic resources, and large-scale adoption of breeding and intensification processes^6^. In particular, the large number of imported breeds, and their wide promotion, have greatly threatened Chinese domestic animal genetic resources^7^. As examples, the Guping chicken, Lintao chicken, Wenshan goose, and Simao goose have all become extinct and, in total, 44 breeds are on the edge of extinction and 15 are endangered^8,9^. In response to this crisis, the departments responsible for managing Chinese animal genetic resources have allocated yearly funds toward conservation efforts. Since these funds are limited, both conservation strategy and fund allocation are determined by the economic value and population size of a breed^10^. However, the subjectivity of this system could result in ineffective conservation of precious and endangered genetic resources. Therefore, a better system is needed to determine priority in these conservation efforts and achieve optimal allocation of funds^11,12^.

One option is through marginal diversity, which was defined by Weitzman in 1992^13^ as a mechanism for measuring genetic diversity. The concept uses genetic and non-genetic factors to calculate a “maximum-likelihood tree”^14^ and the current diversity of breeds, and estimates the expected variations in diversity over a certain time. This approach defines criteria of diversity and relies on quantitative assessments of different strategies, providing concrete reasoning for breed conservation. At present, marginal diversity has been applied in studies on European pigs^15^ and cows^16^; however, no systematic assessment with this approach has been conducted in Chinese domestic duck breeds and the managers also do not know how to allocate funds for breed insurance. Here, we use short tandem repeat profiling to perform a marginal diversity analysis of 21 Chinese domestic duck breeds or genetic resources, which can be used to determine conservation priority.

## Materials and Methods

All animal experiments were performed in accordance with the Regulations for the Administration of Experimental Animals issued by the Ministry of Science and Technology (Beijing, China). All experiments were approved by the Animal Care and Use Committee of Yangzhou University.

### Breed and genetic distance measurements

The objects of the study were Chinese domestic duck breeds or their genetic resources. Their name, sample size, and origin are shown in Table 1. Blood was collected according to pedigree, to ensure that samples were from unrelated individuals. The samples (0.5 mL) obtained from the vein of the ducks wings were carefully mixed with lysis solution and kept at 4 °C for subsequent DNA extraction. DNA extraction was performed according to the method described by Huang *et al*.^17^.

**Table 1:** Name, sample size, and origin of 21 Chinese domestic duck breeds.

Breed	Abbreviation	Sample size	Economic use	Feather color	Existing quantity	Origin
Beijing duck	BJ	96	meat	white	49,900,000	Jade Spring Hill, Beijing
Chaohu duck	CH	80	meat/egg	hemp	2,000,000	Lujiang, Chaohu, Anhui
Dayu duck	DY	96	meat	hemp	110,000	Dayu, Ganzhou, Jiangxi
Chinese Muscovy duck (Chinese Fanya)	FY	96	meat	white/black	1,200,000	Honduras
Guangxi small sheldrake	GX	72	egg/meat	hemp	10,000,000	Xilin, Baise, Guangxi
Gaoyou duck	GY	66	egg/meat	hemp	2,000,000	Gaoyou, Jiangxi
Ji’an red duck	JA	80	meat/egg	brown red	10,000,000	Suichuan, Ji’an, Jiangxi
JIanchang duck	JC	96	meat/egg	hemp	530,000	Xichang City and Dechang County, Sichuan
Jinding duck	JD	80	egg	hemp	12,000	Zini, Longhai, Fujian
Jingjiang sheldrake	JJ	80	meat	hemp	136,000	Jingzhou, Hubei
Jianshui brown duck	JS	96	meat/egg	brown	12,000	Jianshui, Lin’an, etc., Yunnan
Jingxi large sheldrake	JX	72	egg/meat	hemp	400,000	Jingxi, Baise, Guangxi
Liancheng white duck	LC	96	fancy	white	1,500,000	Liancheng, Longyan, Fujian
Linwu duck	LW	72	egg	light gray hemp	6,510,000	Linwu, Chenzhou, Hunan
Mawang duck	MW	96	egg	light gray hemp	466,000	Youyang, Chongqing
Putian black duck/coot	PT	96	meat/egg	black	150 000	Lingchuan, Putian, Fujian
Shanma (Mountain) duck	SM	72	egg	light gray hemp	25,000	Longyan, Fujian
Sansui duck	SS	96	egg/meat	hemp	10,000	Sansui, Guizhou
Taiwan duck	TW	96	egg/meat	dun	2,400,000	Yilan, Dalin, etc., Taiwan
Youxian County sheldrake	YX	72	egg	light gray hemp	5,800,000	Youxian, Zhuzhou, Hunan
Zhongshan sheldrake	ZS	96	meat/egg	hemp	None	Zhongshan, Guangdong

Twelve pairs of microsatellite primers with rich polymorphism were selected as follows: APH01, APL2, AJ272579, AJ272578, AJ272577, AJ415887, AJ515884, AJ515893, AY493256, AY493289, AY493313, and CMO11. The Sequences, combination, and optimal reaction condition have been reported previously^18^. A total of 1802 ducks were genotyped and the population genetic parameters calculated were described in a published paper^18^. The standard genetic distance between populations was calculated with Microsatellite-Toolkit^19^ and Dispan (http://www.softpedia.com/get/Science-CAD/DISPAN.shtml).

### PCA and population structure analysis for all breeds

In this study, SPSS13.0 software was used for principal component analysis (PCA) of all the detected alleles^20^, and Structure 2.0 (http://rosenberglab.bioinformatics.med.umich.edu/distruct.htm) software was used for genetic Structure analysis of 21 populations.

### Extinction probability

Extinction probability is an important index for genetic resource diversity. Future changes in the diversity of local breeds or genetic resources can be measured as the extinction probability over time (500 years)^21^. In Weitzman’s approach, the extinction probability (Zi) of each set is a variable that needs special attention. There are various methods for calculating Zi; however, we adopted the method proposed by Reust-Marti^11^. This method uses seven variables: the total population size (POS), its change over the past 10 years (CHA), distribution of the breed (DIS), risk of indiscriminate crossing (CRO), organization and conservation measures of breeding (ORG), special traits (SPE), and threat of production transition (PRO) (Table 2). Different weights (w_*i*_) were given to different variables to estimate Zi in the future 500 years. The estimate formula and correction formula are as follows:

1 $$Z{\rm{i}}=\mathop{\sum }\limits_{i=1}^{n}{{\rm{w}}}_{i}{{\rm{x}}}_{i}/\mathop{\sum }\limits_{i=1}^{{\rm{n}}}{\rm{\max }}{{\rm{w}}}_{i}{{\rm{x}}}_{i}$$

where, w_*i*_ is the weight of each variable (w_1_ = 0.35, w_2_ = 0.15, w_3_ = 0.14, w_4_ = 0.10, w_5_ = 0.10, w_6_ = 0.06 and w_7_ = 0.10) and x_*i*_ is the estimate of the i^th^ indicator. The seven parameters for this analysis were attained by on-site observation, literature review, and estimation, in order to calculate the Zi of each breed and genetic resource in the next 500 years. For the convenience of calculation, Zia of each breed or genetic resource was corrected to 0.1–0.9, according to the formula below^22^.

2 $$Zi=\frac{0.8}{1.2}\times \mathop{\sum }\limits_{a=1}^{7}Zia+0.1$$

**Table 2:** Influencing factors and criteria of extinction probability.

Influencing factor	Abbreviation	Grading standard
Total population size	POS	0.3 < ten thousand; 0.2 = ten thousand to one hundred thousand; 0.1 = one hundred thousand to one million; 0 = one million
Change of total population size over the past 10 years	CHA	0.1 = decreasing (>20%); 0 = increasing or maintaining stability
Distribution of the breed	DIS	0.2 = county; 0.1 = city; 0 = trans-regional and trans-provincial areas
Risk of indiscriminate crossing	CRO	0.2 = high degree; 0.1 = moderate degree; 0.05 = low degree; 0 = No
Organization and conservation measures of breeding	ORG	0.2 = No; 0 = Yes
Special traits	SPE	0.1 = None; 0 = Yes
Threat of production transition	TRA	0.3 = high degree; 0.2 = moderate degree; 0.1 = low degree

For the set (S) containing a certain number (N) of breeds and genetic resources, and a breed *i*, the distance of j ∈ S can be expressed as d_*ij*_. According to Weitzman’s recursive algorithm, the diversity variable D (S) can be calculated by an N × N distance matrix. The probability of a breed’s existence in 500 years is 1-Zi, if Z is an N-dimensional vector containing Zi of N sets. K is an N-dimensional vector containing the indicator variable Ki (i = 1, 2, … N). Ki = 1 if the set *i* exists, whereas Ki = 0 if the set *i* is extinct. Therefore, K represents an overview of the status in which a subset of breeds exists and its complementary subset is extinct. The formula of the existence probability of a subset of breeds is as follows:

3 $$P(K)=\prod _{{\rm{i}}}({\rm{Ki}}+{(-1)}^{{{\rm{k}}}_{{\rm{i}}}}{{\rm{z}}}_{{\rm{i}}})$$

D_K_ is the diversity of the subsets safe from extinction. The expected diversity at the end of the time horizon (500 years) is calculated as:

4 $$E(D)=\sum _{\forall K}P(K){D}_{K}$$

The variance of the expected diversity is:

5 $${{\rm{D}}}_{{\rm{i}}}^{^{\prime} }=\frac{\partial E(D)}{\partial {z}_{i}}V{\rm{ar}}({\rm{D}})=\sum _{\forall K}P(K){D}_{K}^{2}-{[\sum _{K}P(K)DK]}^{2}$$

The marginal diversity of a breed or genetic resource reflects the variation of the expected diversity when the extinction probability is increased by one unit. The marginal diversity is calculated as follows:

6 $${{\rm{D}}}_{{\rm{i}}}^{^{\prime} }=\frac{\partial E(D)}{\partial {z}_{i}}$$

Based on the extinction probability and expected diversity of a breed or genetic resource, Weitzman suggested conservation potency as the optimal parameter to assess the genetic diversity over a given time horizon. The conservation potency (CP) is calculated as follows:

7 $$C{P}_{i}={z}_{i}\times {D}_{i}^{^{\prime} }$$

*CP*_*i*_ represents a possible increase in the expected diversity of a breed or genetic resource when the threat is completely removed. According to previous work by Simianer *et al*.^10^, *CP*_*i*_ is the optimal parameter for determining conservation schemes, with the highest *CP*_*i*_ *requiring a* minimum amount of capital required for the protection scheme^22^. The breed or genetic resource with the highest *CP*_*i*_ should be allocated the least funds in breeding conservation efforts^10,23^.

## Results

### Genetic distance

**Figure 1:**
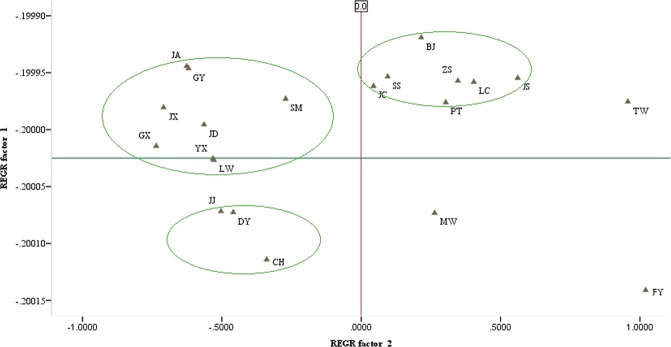
Two-dimensional scatter plot of the first and second factors for 21 duck populations. Note: Breed abbreviations are defined in Table 1.

Twelve simple sequence repeats were detected in the 21 duck breeds. Nei’s standard genetic distance was estimated using Microsatellite-Toolkit and Dispan^24^. According to the genetic distance matrix (Table 3) and cluster analysis by the unweighted pair-group method with arithmetic means (Fig. 1)^25^, FY belonging to the *Cairina* breed forms a single set, whereas the other 20 duck breeds (*Anas*) form three large sets. The distance between GY and JA was the shortest, and the distance between FY and GX was the longest First bullet.

**Table 3:** Nei’s standard genetic distance between 21 Chinese domestic duck breeds.

	BJ	CH	DY	FY	GX	GY	JA	JC	JD	JJ	JS	JX	LC	LW	MW	PT	SM	SS	TW	YX
CH	0.3849																			
DY	0.3552	0.2093																		
FY	1.4498	1.5928	1.7169																	
GX	0.5577	0.5698	0.2720	2.3350																
GY	0.4749	0.3001	0.2800	2.0529	0.1984															
JA	0.5655	0.4483	0.3074	2.1508	0.1557	0.0700														
JC	0.1875	0.4740	0.3797	1.4489	0.5702	0.6435	0.7541													
JD	0.5225	0.3202	0.2559	1.9372	0.1793	0.1212	0.1301	0.6755												
JJ	0.3738	0.2097	0.1989	1.7176	0.2734	0.1910	0.2287	0.4072	0.1355											
JS	0.2040	0.3825	0.4276	1.3069	0.6836	0.5397	0.6659	0.2801	0.5232	0.4767										
JX	0.3935	0.4419	0.1989	2.0945	0.2999	0.4227	0.4163	0.3683	0.3702	0.2440	0.4795									
LC	0.5355	0.5450	0.4381	1.2576	0.7158	0.6798	0.7618	0.4471	0.7652	0.5228	0.5866	0.4956								
LW	0.4433	0.3063	0.2631	2.1002	0.3364	0.3405	0.3244	0.4824	0.2751	0.2529	0.5010	0.3217	0.7047							
MW	0.2260	0.3622	0.3898	1.2259	0.6588	0.5169	0.6061	0.3178	0.4908	0.4347	0.2043	0.4445	0.3674	0.4905						
PT	0.6262	0.6331	0.5018	1.2362	0.7920	0.7392	0.8338	0.5651	0.6991	0.5654	0.5596	0.4819	0.1791	0.5982	0.4644					
SM	0.4500	0.3127	0.2506	1.7219	0.3043	0.2494	0.2266	0.6413	0.1110	0.2022	0.4304	0.4134	0.5293	0.4080	0.3766	0.6702				
SS	0.2863	0.2854	0.2604	1.4692	0.4835	0.3242	0.4179	0.3951	0.3245	0.2849	0.3223	0.3351	0.2986	0.3026	0.1489	0.3283	0.3262			
TW	0.3868	0.6315	0.5551	1.2674	0.8891	0.8208	0.8472	0.5227	0.7868	0.6760	0.4128	0.6160	0.4060	0.7507	0.1730	0.3802	0.5840	0.3922		
YX	0.4048	0.2970	0.1567	1.8717	0.3296	0.3464	0.3301	0.4498	0.3180	0.3098	0.4438	0.3354	0.5949	0.1912	0.4687	0.5947	0.2805	0.3386	0.6838	
ZS	0.1228	0.3896	0.3505	1.4752	0.5732	0.5210	0.5913	0.1998	0.4878	0.3876	0.2101	0.3239	0.4621	0.4121	0.1372	0.5241	0.4182	0.2113	0.3126	0.4576

Note: Breed abbreviations are defined in Table 1.

### PCA and population structure analysis for all breeds

PCA was performed on the gene frequencies of all alleles detected in 12 SSR seats in 21 populations. The plane distance graph constructed according to the first two principal components was shown in Fig. 1. 21 duck breeds were divided into three large groups. Among them, BJ, ZS, SS, JC, PT, JS, LC were relatively close to each other. JJ, DY and CH were close to each other, forming another group. In addition, 9 breeds including JA, GY, SM, JX, JD, GX, YX, LW constituted a group. The distances between FY and other 20 domestic duck breeds were relatively large.

**Figure 2:**
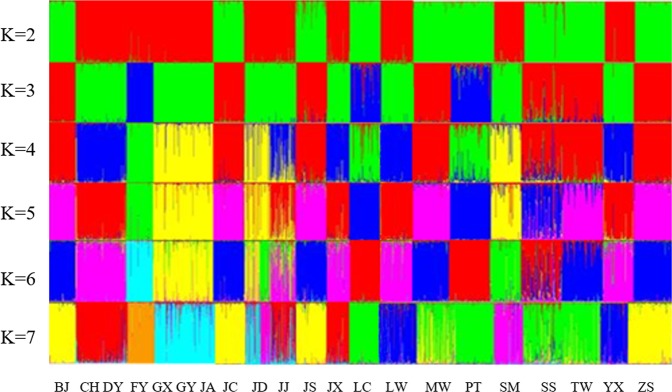
Population structure of 21 populations by the individual Q matrix structure. (Running Structure 1000 times from K = 2 to 7). Note: Breed abbreviations are defined in Table 1.

Structure 2.0 program can group individuals with similar genotypes on multiple SSR seats without prior knowledge of the fusion or evolutionary history of the populations (Fig. 2). When K = 2, no single population was isolated; Of these, 9 breeds such as BJ, JC, JS, LC, MW, PT, SS, TW, ZS were grouped together, and the rest of 12 breeds were clustered into one group. When K = 3 and K = 4, no single group was isolated. FY, LC, PT were clustered into one group. FY ducks were isolated as a single group until K = 5. When K = 7, BJ, JC, JS and ZS still formed a group.CH, DY and JX still gathered into one group: GX, GY, JA, JD CLUSTERED into the last one group.

### Extinction probability

The average extinction probability for the 21 Chinese domestic duck breeds and genetic resources was 0.38% (Table 4). ZS, GX, and JA had the largest extinction probabilities, whereas LW, JD, and GY had the smallest.

**Table 4:** Extinction factor weighting and extinction probability correction of each population.

Breed name	POS	CHA	DIS	CRO	ORG	SPE	TRA	Zi	Correction
Weight	0.35	0.15	0.14	0.10	0.10	0.06	0.10	1.00	
BJ	0.00	0.00	0.00	0.20	0.00	0.00	0.30	0.05	0.23
CH	0.20	0.00	0.10	0.20	0.20	0.10	0.20	0.15	0.48
DY	0.30	0.00	0.20	0.10	0.20	0.10	0.20	0.19	0.58
FY	0.10	0.00	0.00	0.00	0.20	0.10	0.10	0.07	0.28
GX	0.30	0.10	0.10	0.05	0.20	0.10	0.30	0.20	0.59
GY	0.00	0.00	0.10	0.10	0.00	0.00	0.10	0.03	0.19
JA	0.30	0.10	0.20	0.20	0.00	0.10	0.20	0.19	0.59
JC	0.30	0.00	0.10	0.20	0.00	0.00	0.10	0.15	0.48
JD	0.00	0.00	0.00	0.20	0.00	0.00	0.10	0.03	0.18
JJ	0.20	0.10	0.10	0.10	0.00	0.10	0.20	0.14	0.44
JS	0.20	0.10	0.10	0.20	0.20	0.10	0.30	0.18	0.54
JX	0.10	0.00	0.10	0.20	0.20	0.10	0.30	0.13	0.42
LC	0.20	0.00	0.00	0.20	0.00	0.00	0.10	0.10	0.35
LW	0.00	0.00	0.00	0.10	0.00	0.00	0.10	0.02	0.15
MW	0.10	0.00	0.00	0.20	0.20	0.10	0.10	0.09	0.33
PT	0.10	0.10	0.20	0.10	0.00	0.00	0.30	0.12	0.40
SM	0.00	0.00	0.00	0.20	0.20	0.10	0.20	0.07	0.27
SS	0.10	0.10	0.10	0.20	0.00	0.10	0.20	0.11	0.38
TW	0.00	0.00	0.10	0.05	0.20	0.10	0.10	0.06	0.24
YX	0.00	0.00	0.00	0.10	0.00	0.10	0.20	0.04	0.19
ZS	0.30	0.10	0.20	0.20	0.20	0.10	0.30	0.22	0.67

Breed abbreviations are defined in Table 1.

### Current and expected future diversity

The current diversity of the 21 breeds and genetic resources was determined to be 7.72, and the expected diversity of all sets in 500 years is 5.14 ± 1.15. Therefore, an overall decrease of 2.58 (33.43%) is anticipated.

### Contributions and marginal diversities of each breed

**Figure 3:**
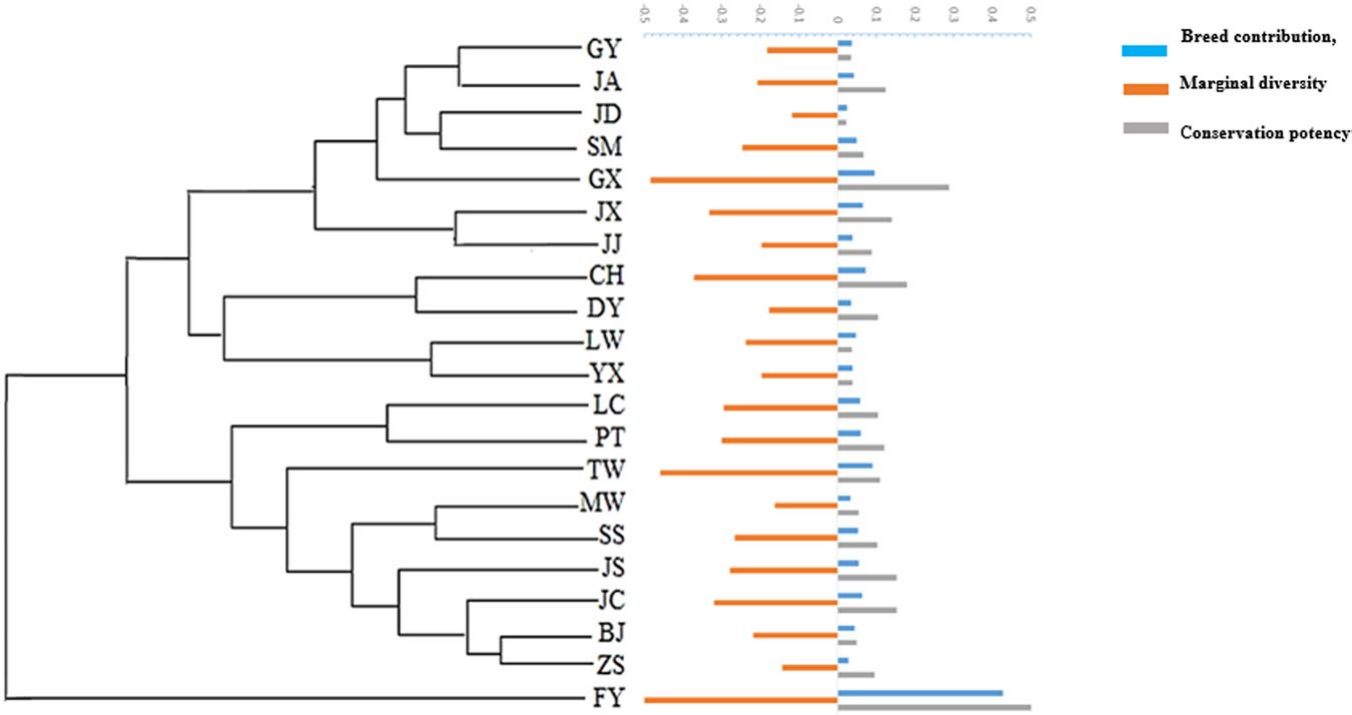
A maximum-likelihood tree showing the marginal diversity, contribution, and conservation potency of each breed. Note: Breed abbreviations are defined in Table 1.

The term “contributions” is defined as the percentage of contribution of each breed to overall diversity. The contributions and marginal diversities of each of the 21 breeds are shown in Table 5 and Fig. 3. The contribution of FY was the largest (Table 3) followed by GX, whereas JD had the smallest contribution. Similarly, in terms of marginal diversity, FY showed the largest (Table 5), followed by GX, whereas JD has the smallest (−0.12, 2.32). Finally, FY had the highest conservation potency, followed by GX, and JD had the lowest.

**Table 5:** Marginal diversity of 21 Chinese domestic duck breeds.

Breed name	Extinction probability	Contribution (%)	Marginal diversity	Conservation potency
BJ	0.23	4.25	−0.2183	0.0494
CH	0.48	7.26	−0.3732	0.1789
DY	0.58	3.47	−0.1783	0.1031
FY	0.28	42.61	−2.1896	0.6120
GX	0.59	9.44	−0.4849	0.2876
GY	0.19	3.57	−0.1834	0.0341
JA	0.59	4.07	−0.2091	0.1235
JC	0.48	6.23	−0.3202	0.1527
JD	0.18	2.32	−0.1192	0.0210
JJ	0.44	3.85	−0.1977	0.0872
JS	0.54	5.43	−0.2790	0.1513
JX	0.42	6.49	−0.3335	0.1388
LC	0.35	5.75	−0.2955	0.1043
LW	0.15	4.63	−0.2381	0.0359
MW	0.33	3.18	−0.1633	0.0539
PT	0.40	5.87	−0.3015	0.1201
SM	0.27	4.80	−0.2469	0.0659
SS	0.38	5.22	−0.2682	0.1014
TW	0.24	8.95	−0.4597	0.1099
YX	0.19	3.85	−0.1977	0.0378
ZS	0.67	2.79	−0.1434	0.0955

Breed abbreviations are defined in Table 1.

## Discussion

There are numerous domestic duck breeds in China including 27 indigenous breeds, two introduced breeds, and a few developing breeds. However, with the introduction and promotion of cherry valley duck, the number of local duck species in China has dropped sharply, and many species are facing the danger of extinction^26^. Conservation of genetic diversity plays an important role in sustaining the livestock breeds^27^. At present, Weitzman marginal diversity method has attracted more and more attention in the research on rational allocation of livestock and poultry resources protection funds, and has become one of the most dynamic theories in the field of livestock genetic resources protection and utilization^28,29^. There were many researches on animal genetic diversity in the world^30–32^, but few of them analyze the application of marginal diversity method To our knowledge, Reist-Marti *et al*.(2003) have estimated extinction probability in livestock breeds^11^. Bennewitz (2005) estimated the extinction probabilities of 5 German dual-purpose cattle breeds by population viability analysis^33^. And then (2006) he analyzed 44 North Eurasian cattle breeds using simplified determined extinction probabilities. The results show that the expected loss of diversity within the next 50 years is between 1 and 3% of the actual diversity^34^. The marginal diversity analysis of goat^29^, sheep^35^, cattle^36^, pigs^37^ has been completed in China, which provides a reliable data reference for the division of conservation funds. After years of investigation of domestic duck resources and collection of blood samples, this paper analyzed the marginal diversity and extinction probability of local duck breeds in China for the first time.

In this paper, the PCA was used to explain the molecular genetic relationships among the populations and a plane distance map was constructed, reflecting the real genetic information and genetic relationships of the 21 populations. Structure cluster analysis use allelic and genotype data from multiple loci, such as SSR loci, to construct a cluster model. Structure 2.0 program was based on Bayesian probability theory, adopt Markov-Monte Carlo simulation algorithm, and used mixed model when running the program to reveal the unknown population genetic relationship and potential population Structure from all population levels^38^. The expected number of classification (K value) of the detected group was set at runtime, which can be used to divide all individuals and reflect the genetic structure of the group. It is especially suitable for the study of the genetic structure, the differentiation and migration of individuals. In this paper, the population Structure diagram and the maximum-likelihood tree^39^ obtained based on Structure 2.0 program were consistent with the results from PCA, verifying the accuracy of population Structure inference.

The calculation for extinction probability considers all factors that might cause change in a breed or genetic resource, making it an accurate and reliable estimation^40^. However, due to the political and economic situation in China, as well as the distribution of indigenous duck breeds and resources, some factors were not considered in this study, such as natural disasters, reliability of the information source, and development of reasonable storage approaches. For this measurement, seven variables (POS, CHA, DIS, CRO, ORG, SPE, and TRA) were assigned to different weights as major factors. These variables have been proved to be important factors reflecting population diversity. Here, we calculated the current and expected population diversities of a total of 21 Chinese duck breeds, respectively. Importantly, we found that the expected diversity (within 500 years) were 33.43% lower than current diversity.

Some variables for calculating the extinction probability, such as CHA and CRO, only consider the conservation of a single breed or genetic resource, and do not account for the effect of the conserved breed on the genetic diversity of the entire population^41^. If limited breeding conservation funding is allocated based on extinction probability parameters, it may not be the most beneficial solution for the entire population, especially if that population includes numerous breeds and strains. Instead, the breed with the largest contribution should be given the highest priority^42,43^. In this study, the largest contributor was FY, followed by GX.

However, breed contribution is not the only consideration for conservation planning efforts, and its calculation does not consider extinction probability. In contrast, marginal diversity considers both contribution and the extinction probability, and can therefore act as a comprehensive measurement of the importance of each breed. According to Weitzman, marginal diversity parameters should be considered the preferred reference during breeding conversation planning^44^. Conserving the breed with the largest conservation potency is the most effective way to maintain overall genetic diversity. Therefore, the first two breeds prioritized should be FY and GX, followed by CH, JC, JS, JX, JA, PT, TW, LC, DY, SS, ZS, JJ, SM, MW, BJ, YX, LW, GY, and JD.

Based on the marginal diversity parameters, we identified the conservation priority of 21 local duck breeds and genetic resources. FY and GX are the first two breeds that should be protected. The conservation priority in this study can provide a reference for breed conservation planning.

## Data Availability

The data generated and analyzed as a part of this study are included within this article.
